# Plasma thrombin-antithrombin complex as a candidate biomarker for coronary slow flow

**DOI:** 10.3389/fcvm.2025.1621655

**Published:** 2025-07-15

**Authors:** Jian-hong Mo, Bo Liang, Jing-tao Cen, Wan-ying Li, Yong-an Mo, Ming-cheng Tang, Dong Ye, Qiu-xia Long, Xun Hu, Yuan-sheng Zhai

**Affiliations:** ^1^ Department of Cardiology, the People’s Hospital of Fengkai County, Zhaoqing, Guangdong, China; ^2^ Department of Cardiology, the First People’s Hospital of Zhaoqing, Zhaoqing, Guangdong, China; ^3^ Department of Cardiology, the First Affiliated Hospital, Sun Yat-Sen University, Guangzhou, China; ^4^ Key Laboratory on Assisted Circulation, Ministry of Health, Guangzhou, China

**Keywords:** thrombin-antithrombin complex, coronary slow flow, biomarker, atherosclerosis, thrombolysis in myocardial infarction frame count

## Abstract

**Background:**

Coronary slow flow (CSF), characterized by delayed coronary perfusion without significant coronary artery stenosis, remains a diagnostic challenge due to its elusive pathophysiology. This retrospective study aimed to evaluate the association between the thrombin-antithrombin (TAT) complex and CSF.

**Patients and methods:**

Ninety-one CSF patients and 74 subjects with normal coronary flow were recruited in this cohort. Coronary artery blood flow was quantified using the thrombolysis in myocardial infarction frame count (TFC) method. Plasma TAT complex levels were measured via chemiluminescent immunoassay. Logistic regression analyses and a receiver operating characteristic (ROC) curve were performed to determine the predictive value of TAT for CSF.

**Results:**

Compared with patients without CSF, patients with CSF showed higher plasma levels of TAT complex, total cholesterol, and low-density lipoprotein cholesterol, all of which were also positively correlated with TFC. However, multivariate logistic regression identified TAT as the only independent predictor of CSF after adjustment (OR: 1.71, 95% CI: 1.39–2.10, *p* < 0.001). More specifically, ROC analysis revealed that a plasma TAT complex levels of 3.875 ng/ml predicted CSF with a specificity of 89.2% and a sensitivity of 62.6%.

**Conclusion:**

Elevated plasma TAT complex levels are strongly associated with CSF and may serve as a candidate diagnostic biomarker.

## Introduction

In 1972, Tambe et al. first described the phenomenon of coronary slow flow (CSF) in six patients presenting with chest pain (1). CSF is characterized by the delayed perfusion of distal vessels despite the absence of significant coronary artery stenosis (stenosis <40%) on coronary angiography (CAG). The incidence of CSF ranges from 0.2% to 7% in routine CAG patients, but increases to 25% in those with chest pain (2–4). While the left anterior descending artery (LAD) is most frequently involved, followed by the left circumflex (LCX) and right coronary arteries (RCA), over 80% of CSF patients experience recurrent precordial discomfort, chest pain, or angina (5–7). Although the majority of patients with CSF tend to have a favorable prognosis, a minority of cases may experience acute myocardial infarction, life-threatening arrhythmias, and sudden death, thus requiring adequate attention from clinicians (8, 9).

While most studies report no significant differences in atherosclerotic risk factors between CSF patients and those with normal coronary flow, some recent studies have specifically linked CSF to elevated total cholesterol (TC), increased low-density lipoprotein cholesterol (LDL-C) levels (10). However, the pathogenic mechanism of CSF remains multifactorial and incompletely elucidated. Extensive studies suggest that possible mechanisms may include endothelial dysfunction in the coronary arteries, microvascular dysfunction, inflammation, genetic factors, subclinical atherosclerosis and hemorheological abnormalities (11). Research has demonstrated that patients with CSF have notable calcification in the coronary vessel walls, diffuse thickening of the intima, and non-obstructive changes in the coronary arteries due to atherosclerosis, therefore suggesting that patients with CSF may have undergone subclinical atherosclerotic alterations (12, 13). Hemorheological abnormalities, including increased blood viscosity, decreased erythrocyte deformability, and heightened platelet aggregation, play a critical role in the development and progression of coronary atherosclerosis, which may contribute to CSF (14, 15). Korhan et al. recently found that erythrocyte aggregation caused CSF and may be a new treatment target for CSF patients (16). Furthermore, it has been demonstrated that patients with CSF exhibit significantly elevated mean platelet volume, a marker indicative of platelet activation and platelet aggregability, in comparison with patients with normal coronary flow (17). These hemorheological factors may impair coronary microcirculatory flow, exacerbating ischemia despite macrovascular patency. Emerging evidence indicates that an imbalance in coagulation activity, often reflected by biomarkers such as thrombin-antithrombin (TAT) complex, may further link hemorheological disturbances to CSF.

Thrombin, a serine protease central to coagulation, converts fibrinogen to fibrin and activates platelets via protease-activated receptors (PARs) (18). Its inactivation through irreversible binding to antithrombin forms TAT complexes, a validated marker of thrombin generation (19). Beyond its canonical role in hemostasis, thrombin exerts pleiotropic effects on cellular processes, including exacerbation of endothelial dysfunction, induction of vascular permeability, promotion of oxidative stress, triggering of apoptotic pathways, and upregulation of pro-inflammatory cytokines (20). These multifunctional actions highlight its critical involvement in vascular pathophysiology. Emerging evidence underscores the role of thrombin in the pathogenesis of coronary atherosclerosis. Direct evidence supporting thrombin's involvement in atherogenesis derives from experimental studies demonstrating that pharmacological inhibition of thrombin attenuates atherosclerosis progression in apolipoprotein E-deficient mice (21). Julian et al. further demonstrated that early-stage atherosclerotic lesions exhibited both enhanced thrombin generation capacity and markedly elevated TAT complex levels (22). Clinical studies indicate that elevated TAT levels are significantly associated with the presence and severity of coronary artery disease (CAD) (23). Notably, patients with acute myocardial infarction have higher peak thrombin generation rates compared to those with stable CAD, as quantified by TAT complex levels (24). However, the association between TAT complex and CSF remains unclear.

At present, CAG remains the only reliable way of detecting CSF. Given its expense and invasiveness, it's crucial to develop cost-effective, noninvasive diagnostic alternatives for CSF. To address this gap, the current study aims to determine the potential clinical value of TAT complex as a candidate circulating biomarker for CSF screening.

## Patients and methods

### Study design and participants

This retrospective cohort study utilized patient data from individuals undergoing selective coronary artery angiography at the People's Hospital of Fengkai County during the period spanning May 2023 to October 2024. From the institutional medical archives, we systematically identified 724 eligible cases that met the following inclusion criteria: (1) over 18 years old; (2) completion of diagnostic coronary angiography; and (3) availability of complete medical documentation encompassing demographic profiles, clinical histories, plasma TAT complex levels and procedural records. Exclusion criteria included: (1) previous coronary revascularization (percutaneous coronary intervention or coronary artery bypass graft surgery); (2) coronary artery ectasia or spasms; (3) concomitant structural cardiac pathologies (e.g., cardiomyopathy, severe valvular heart disease, congenital heart disease, or severe heart failure); (4) active hematological disorders; (5) Significant renal/hepatic dysfunction; (6) documented thyroid dysfunction; (7) history of malignancy; (8) acute/chronic inflammatory conditions or active systemic infections; and (9) missing TAT levels data; (10) presence of ≥50% luminal narrowing in major epicardial vessels.

**Figure 1:**
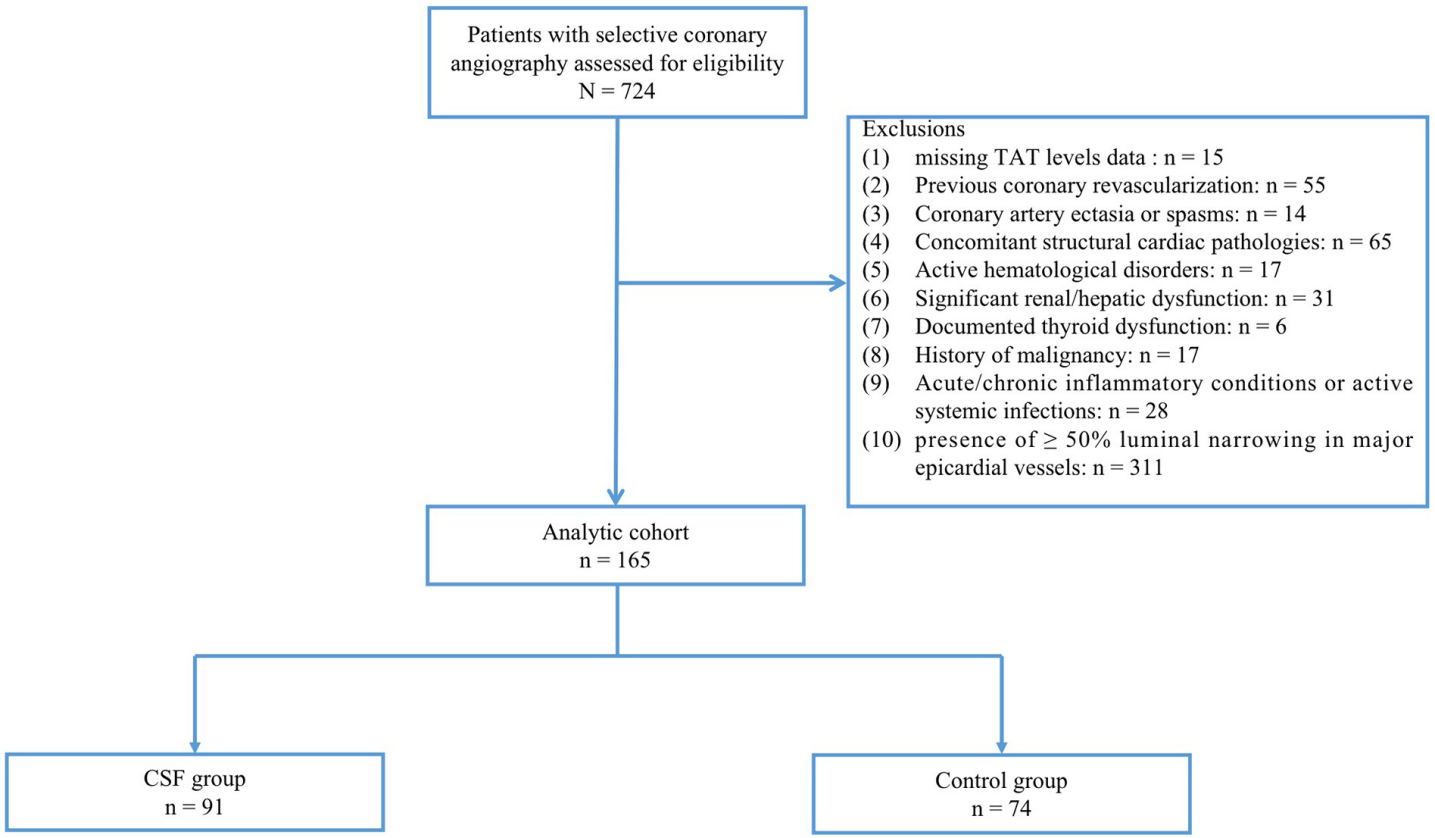
Study flow chart. CSF was defined as TFC > 27 in at least 1 coronary artery despite the absence of visually detectable coronary stenosis or ≥50% luminal narrowing. CSF, coronary slow flow; Control group, patients with normal coronary flow.

The final study population comprised 165 patients with angiographically confirmed absence of visually detectable coronary stenosis or ≥50% luminal narrowing in major epicardial vessels. Participants were divided into two groups: 91 patients exhibiting CSF phenomenon (CSF group) and 74 controls with normal coronary flow (control group) (Figure 1). Coronary artery blood flow was quantified using the thrombolysis in myocardial infarction (TIMI) frame count (TFC) method, with CSF defined as TFC >27 in at least 1 coronary artery. This study was conducted in strict accordance with the Declaration of Helsinki and approved by the ethics committee of the local hospital. Given the retrospective nature of this study, verbal informed consent was approved by the ethics committee.

### Coronary angiography and assessment of TFC

Selective coronary angiography was performed on all participants using the Judkins technique 24 h after venous blood samples were collected to measure TAT complex levels. Coronary visualization was performed in multiple orthogonal projections, including left/right anterior oblique views with cranial and caudal angulations. Quantitative assessment of coronary blood flow was conducted using the TFC method. Angiograms were recorded at 30 frames per second (fps), with TFC defined as the number of cine frames required for contrast medium to propagate from the coronary ostium to standard distal landmarks of the relevant coronary arteries. The initiation frame was defined as the first frame demonstrating complete contrast opacification of the proximal coronary ostium with anterograde laminar flow (≥75% vessel border delineation). The termination frame was determined as the initial visualization of contrast medium reaching standard distal landmarks. These landmarks were defined as follows: (1) LAD: Terminal bifurcation at the cardiac apex; (2) LCX: Distal bifurcation of the segment with the longest total distance; (3) RCA: First branch of the posterolateral artery. As previously described, the LAD frame count was divided by 1.7 to calculate the corrected thrombolysis in myocardial infarction frame count (CTFC), due to the LAD's greater length compared to other major coronary arteries. The average TFC per subject was determined by summing the CTFCs of the LAD, LCX, and RCA, and dividing the total by three. Two independent interventional cardiologists, blinded to clinical data, performed all measurements. Discrepancies were resolved by consultation with a third cardiologist.

### Data collection and study definitions

The following clinical parameters were systematically collected for each enrolled patient: (1) Demographic characteristics: age, gender, height and weight; (2) History of smoking and alcohol use; (3) Medical comorbidities: documented diagnoses of hypertension, diabetes mellitus, and prior coronary revascularization procedures; (4) Biochemical profiles: TAT complex levels, homocysteine concentration, lipid panel [triglycerides (TG), TC, LDL-C], uric acid levels, alanine aminotransferase (ALT); (5) Transthoracic echocardiographic parameters; (6) Coronary angiographic findings.

Blood pressure measurements were obtained following standardized protocols using a validated electronic sphygmomanometer. Measurements were recorded three times after patients rested in a seated position for 5 min, with the mean value used for statistical analysis. According to current clinical guidelines, hypertension was defined as systolic blood pressure (SBP) ≥140 mmHg and/or diastolic blood pressure (DBP) ≥90 mmHg, a documented history of antihypertensive medication use, or self-reported hypertension (25).

Diabetes mellitus was diagnosed on the basis of current clinical guidelines, fulfilling at least one of the following criteria: (a) fasting plasma glucose ≥126 mg/dl (7.0 mmol/L); (b) 2-h postprandial plasma glucose ≥200 mg/dl (11.1 mmol/L) during standardized 75 g oral glucose tolerance testing; (c) glycated hemoglobin ≥ 6.5%; (d) physician-documented diagnosis of type 2 diabetes mellitus in medical records with concurrent antihyperglycemic therapy (26).

### Biochemical parameters assays

Venous blood samples were obtained from all participants after a 12-h overnight fast following hospital admission. All specimens were processed according to standardized laboratory protocols. Plasma TAT complex levels were quantified using chemiluminescent immunoassay (Automated Immunoassay Analyzer, Guangzhou Wondfo Biotechnology Co., Ltd, China) prior to CAG (TAT normal reference range: 0–4 ng/ml). Plasma biomarkers, including complete blood count, lipid profile, fasting glucose, and creatinine, were analyzed with an automated biochemical analyzer.

### Echocardiography

Transthoracic echocardiography was performed with commercially available ultrasound equipment (Mindray Medical International Co., Ltd, Shenzhen, China) by certified sonographers blinded to participant allocation. Left ventricular parameters—including end-diastolic diameter (LVEDD), end-systolic diameter (LVESD), and ejection fraction (LVEF)—were quantified in accordance with the American Society of Echocardiography guidelines (27).

### Statistical analysis

Continuous variables were presented as mean ± standard deviation (for normally distributed data) or median (interquartile range) (for non-normally distributed data), with distribution normality assessed using the Kolmogorov–Smirnov test. Categorical variables were presented as numbers (percentages). The Student's *t*-test or Mann–Whitney *U* test was used to assess group differences for continuous variables, and categorical variables were compared via chi-squared tests. The correlation between mean TFC and TAT complex was analyzed using Pearson correlation test or Spearman's rank correlation test, depending on normality of the distribution. Multivariate logistic regression models were constructed to evaluate associations between CSF and plasma TAT complex levels, with the calculation of odds ratios (ORs) and 95% confidence intervals (CIs). To identify predictive cut-off values of plasma TAT complex for CSF, receiver operating characteristic (ROC) curve analysis was performed. All analyses were performed using SPSS 26.0 (IBM Corporation, Armond, New York, NY, USA), with statistical significance defined as a two-tailed *p*-value <0.05.

## Results

### Demographic and clinical characteristics of study participants

The study enrolled 165 consecutive inpatients, comprising 91 patients with CSF and 74 controls with normal coronary flow. Baseline demographic, biochemical, echocardiographic, and angiographic characteristics are summarized in Table 1. No significant differences were observed between the CSF and control groups in terms of age, gender, diabetes mellitus, or hypertension.

**Table 1:** Demographic and clinical characteristics of study participants.

Variables	CSF group	Control group	*p* value
	*n* = 91	*n* = 74	
Age (years)	62.8 ± 9.7	61.6 ± 9.7	0.464
Gender (%)			0.702
Male	47 (51.6)	36 (48.6)	
Female	55 (48.4)	38 (51.4)	
Hypertension (%)	50 (54.9)	48 (64.9)	0.197
Diabetes mellitus (%)	10 (11.0)	9 (12.2)	0.814
Smoking (%)	32 (35.2)	24 (32.4)	0.712
SBP (mmHg)	116 (105, 127)	121 (110, 128)	0.156
DBP (mmHg)	74 (68, 80)	75 (69, 80)	0.901
Plasma biomarkers			
TAT (ng/ml)	4.43 (2.86, 7.33)	1.82 (1.14, 2.88)	<0.001
Homocysteine (umol/L)	11.39 (8.83, 14.64)	11.11 (9.10, 13.31)	0.551
TC (mmol/L)	4.46 ± 1.09	3.98 ± 1.09	0.006
LDL-C (mmol/L)	2.44 ± 0.79	2.07 ± 0.72	0.002
TG (mmol/L)	1.14 (0.85, 1.88)	1.01 (0.74, 1.76)	0.292
uric acid (umol/L)	333.1 (287.4, 384.9)	332.6 (272.8, 392.8)	0.838
Creatinine (umol/L)	68.6 (59.15, 87.45)	74.8 (65.2, 89.33)	0.155
ALT (U/L)	19.10 (14.95, 26.70)	20.40 (14.83, 26.33)	0.815
WBC (*10^9^/L)	6.81 (5.57, 8.24)	6.91 (5.50, 7.85)	0.625
Hemoglobin (g/L)	129 (123, 138.5)	132 (124, 142.3)	0.117
Platelet (*10^9^/L)	248 (217, 301.5)	231 (179.23, 288.5)	0.016
MPV (fL)	10.5 (8.45, 12.0)	9.6 (7.9, 11.5)	0.132
Echocardiographic parameters			
LVESD (mm)	27.0 (26.0, 30.0)	29.0 (26.0, 31.3)	0.094
LVEDD (mm)	43.0 (40.0, 45.0)	44.0 (41.0, 47.0)	0.090
LVEF (%)	63.0 (60.0, 66.0)	62.0 (59.0, 66.0)	0.472
TFC measurements			
Corrected TFC (LAD)	33.5 (28.2, 42.4)	24.7 (20.6, 26.5)	<0.001
TFC (LCX)	30.0 (25.0, 37.0)	20.5 (17.0, 23.0)	<0.001
TFC (RCA)	31.0 (26.0, 39.0)	22.0 (18.0, 24.0)	<0.001
Mean CTFC	31.5 (26.6, 39.5)	22.6 (18.5, 24.5)	<0.001

CSF, coronary slow flow; TAT, thrombin-antithrombin complex; TC, total cholesterol; LDL-C, low-density lipoprotein cholesterol; TG, triglyceride; single-vessel disease group; ALT, alanine aminotransferase; WBC, white blood cell; MPV, mean platelet volume; LVESD, left ventricular end-systolic diameter; LVEDD, left ventricular end-diastolic diameter; LVEF, left ventricular ejection fraction; TFC, thrombolysis in myocardial infarction (TIMI) frame count; LAD, Left anterior descending artery; LCX, Left circumflex artery; RCA, Right coronary artery. Data were expressed as mean ± standard deviation (SD) or median and interquartile range or *n* (%).

Patients with CSF exhibited significantly higher plasma levels of TAT complex [4.43 (2.86, 7.33) ng/ml vs. 1.82 (1.14, 2.88) ng/ml, *p* < 0.001], TC (4.46 ± 1.09 mmol/L vs. 3.98 ± 1.09 mmol/L, *p* = 0.006) and LDL-C (2.44 ± 0.79 mmol/L vs. 2.07 ± 0.72 mmol/L, *p* = 0.002) compared to controls. Other biochemical parameters, including homocysteine, TG, uric acid, creatinine, alanine aminotransferase, hemoglobin, white blood cell count, mean platelet volume, and platelets showed no significant differences between groups.

As expected, CTFC for LAD, LCX, RCA and the mean TFC were found to be significantly higher in patients with CSF than in controls. However, there were no statistically significant differences between the two groups with regard to LVESD, LVEDD and LVEF.

### Influencing factors of CSF

**Figure 2:**
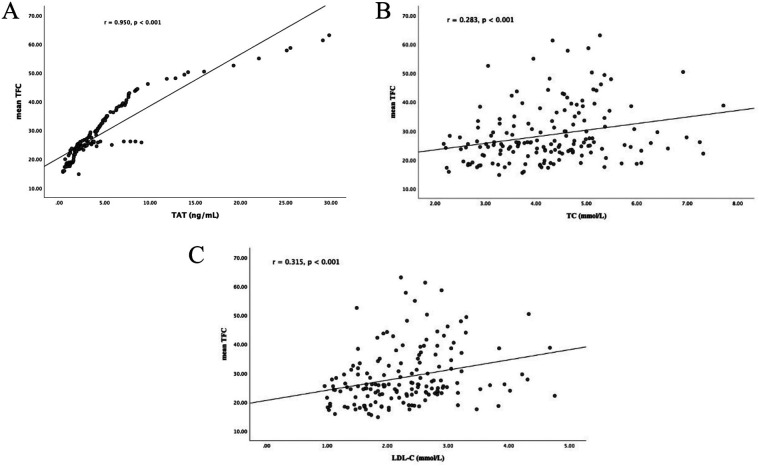
Correlation analyses. **(A)** Correlation between plasma TAT complex levels and mean TFC; **(B)** correlation between plasma TC levels and mean TFC; **(C)** correlation between plasma LDL-C levels and mean TFC. TAT, thrombin-antithrombin; TIMI, thrombolysis in myocardial infarction; TFC, TIMI frame count; TC, total cholesterol; LDL-C, low-density lipo- protein cholesterol.

Plasma TAT complex levels showed a strong positive correlation with mean TFC values among all participants (*r* = 0.950, *p* < 0.001; Figure 2A). Furthermore, elevated mean TFC values were significantly associated with TC (*r* = 0.283, *p* < 0.001; Figure 2B) and LDL-C (*r* = 0.315, *p* < 0.001; Figure 2C).

### Diagnostic predictive value of plasma TAT complex in CSF

Multivariate logistic regression analysis was performed to assess the diagnostic utility of TAT complex levels for CSF. Based on clinical relevance, the multivariate logistic regression model incorporated the following variables: demographic characteristics (age, sex), metabolic parameters (diabetes, hypertension, uric acid, creatinine, homocysteine), hematologic indices (hemoglobin, white blood cell count, platelet count), hepatic function marker (alanine aminotransferase), lipid profiles (TG, TC, LDL-C), and echocardiographic parameters (LVESD, LVEDD and LVEF). After adjustment for these variables, plasma TAT complex levels emerged as an independent predictor of CSF (Table 2). In the fully adjusted model, elevated plasma TAT complex levels was associated with a 1.71-fold increased risk of CSF (OR: 1.71, 95% CI: 1.39–2.10, *p* < 0.001).

**Table 2:** Analysis of factors predicting CSF risk.

Variables	OR	Lower 95% CI	Upper 95% CI	*p* value
TC (mmol/L)				
unadjusted	1.505	1.114	2.032	0.008
adjusted	1.303	0.873	1.944	0.195
LDL-C (mmol/L)				
Unadjusted	1.958	1.257	3.049	0.003
Adjusted	1.592	0.883	2.868	0.122
TAT (ng/ml)				
unadjusted	1.695	1.384	2.076	<0.001
adjusted	1.710	1.391	2.103	<0.001

CSF, coronary slow flow; TC, total cholesterol; LDL-C, low-density lipoprotein cholesterol; OR, odds ratio; CI, confidence interval.

**Figure 3:**
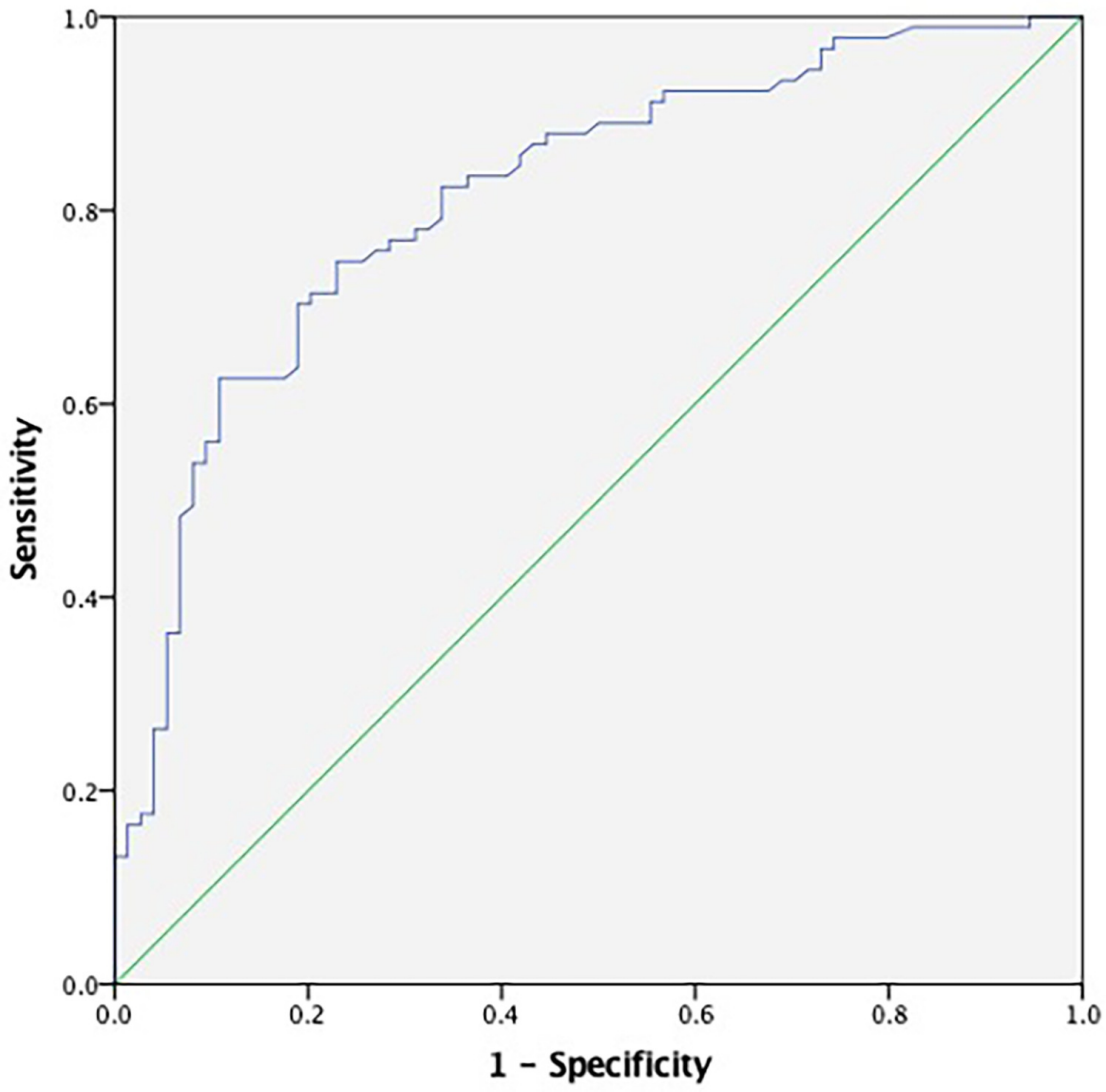
ROC curve analyses for predictive values of plasma TAT complex levels. The ROC curve for TAT demonstrated an area under the curve (AUC) of 0.815 (95% CI: 0.749–0.880, *p* < 0.001), with the blue line representing the diagnostic performance of TAT. TAT, thrombin-antithrombin.

Receiver operating characteristic (ROC) curve analysis demonstrated the diagnostic performance of TAT for CSF. The ROC curve indicated that plasma TAT complex levels could serve as a specific predictor for CSF with an area under the curve of 0.815 (95% CI: 0.749–0.880, *p* < 0.001; Figure 3). Furthermore, our results suggest that a plasma cutoff value of 3.875 ng/ml can effectively distinguish CSF patients from controls, achieving a specificity of 89.2% and sensitivity of 62.6%.

## Discussion

This retrospective study revealed three important findings. First, plasma TAT complex levels were significantly elevated in CSF patients compared to controls, with a strong positive correlation between TAT levels and mean TFC values. Second, multivariate logistic regression identified TAT as an independent predictor of CSF, with elevated TAT levels conferring a 1.71-fold increased risk of CSF. Third, ROC curve analysis established a plasma TAT threshold of ≥3.875 ng/ml for CSF diagnosis, achieving a specificity of 89.2% and sensitivity of 62.6%. To our knowledge, this is the first study to propose TAT as a potential diagnostic biomarker for CSF, offering a noninvasive screening tool for clinical practice.

CSF, underrecognized yet clinically significant cardiovascular disorder, imposes considerable clinical and economic burdens on global healthcare systems. Angiographically defined by delayed distal vessel opacification despite the absence of obstructive coronary artery disease, CSF frequently manifests with symptoms similar to acute coronary syndrome, including recurrent angina and precordial discomfort (28). Although its exact pathophysiology remains elusive, emerging evidence implicates multifactorial mechanisms involving microvascular dysfunction, endothelial impairment, subclinical atherosclerosis, inflammatory responses, and hemorheological disturbances such as impaired erythrocyte deformability and platelet hyperaggregability (11). While CAG remains the diagnostic gold standard, its invasiveness and cost underscore the urgent need for noninvasive biomarkers to facilitate early CSF detection.

Current evidence on CSF risk factors exhibits notable ethnic variability. While some studies report an increased prevalence of hypertension in CSF patients (2, 29), the majority of studies fail to detect any difference in traditional atherosclerotic risk factors between patients with CSF and those with normal coronary flow (30). In alignment with these findings, our study similarly observed no significant differences in age, gender, diabetes, hypertension, or hematologic/biochemical markers (hemoglobin, white blood cell count, platelets, uric acid, homocysteine) between groups. Intriguingly, CSF patients had higher plasma levels of TC and LDL-C, which were weakly associated with elevated mean TFC values. However, multivariate logistic regression confirmed that neither TC nor LDL-C independently predicted CSF, consistent with previous studies (31). In contrast, TAT emerged as the sole independent predictor, highlighting its unique diagnostic value in CSF.

Thrombin, a key enzyme in the coagulation cascade, not only drives fibrin formation but also activates PARs on endothelial cells, promoting inflammation and angiogenesis, and tumor progression (32). Despite its crucial functions, thrombin has a very short half-life and is rapidly inhibited by antithrombin, forming a TAT complex. Thus, this complex serves as a marker of thrombin generation and has been linked to hypercoagulability in various clinical settings, including cirrhosis and cancer. In cirrhosis, increased TAT levels are associated with portal vein thrombosis and mortality (33). Similarly, in cancer patients, elevated TAT levels correlate with an increased risk of thromboembolic events (34). Our findings also showed that elevated TAT levels may independently predict an increased risk of CSF, suggesting that thrombin-driven hypercoagulability, quantified by TAT, may be involve in the development of CSF.

The precise mechanisms through which TAT contribute to CSF remains incompletely elucidated, though current evidence points to several interconnected pathways. First of all, it is postulated that the prothrombotic state, as reflected by elevated TAT levels, could result in microvascular dysfunction, which is a feature of CSF pathophysiology. This hypothesis aligns with clinical trials in acute coronary syndrome populations, where thrombin activity has been strongly linked to patient outcomes, particularly in secondary prevention (35). This suggests that thrombin-driven hypercoagulability, as quantified by TAT, may establish a prothrombotic microenvironment conducive to CSF development. Moreover, thrombin's activation of PARs on endothelial cells triggers the release of pro-inflammatory cytokines and growth factors, fostering vascular inflammation, endothelial dysfunction, and pathological remodeling (36). These thrombin-mediated inflammatory cascades may exacerbate CSF by inducing vasoconstriction and diminishing coronary blood flow. Additionally, higher TAT levels have been associated with the severity of coronary atherosclerosis, which may lead to CSF (23). Atherosclerosis, characterized by plaque buildup within the arterial walls, can cause endothelial dysfunction and decreased arterial compliance, both of which are crucial factors in the development of CSF. A recent study has reported that the coronary arteries in CSF exhibit microvascular dysfunction and diffuse atherosclerosis, which impairs the normal vasodilatory response of the coronary microcirculation (37).

The results of this study have important clinical significance. First, our study identified plasma levels of TAT complex as an independent predictor of CSF, which suggest that TAT has the potential to be a cost-effective, noninvasive screening tool for CSF, especially in settings with limited resources where coronary angiography is not available. In addition, rapid screening reduces unnecessary angiography in primary care settings; Finally, our results challenge the conventional hemodynamic characterization of CSF, proposing its redefinition as a thrombotic-microvascular disorder. This alteration may direct targeted therapies aimed at reducing thrombin activity in high-risk patients.

### Limitations

This study has several limitations. Firstly, its single-center, retrospective design inherently restricts causal inference and introduces potential biases. Secondly, the modest sample size may reduce the statistical power to detect less significant associations. Consequently, a large-scale, multicenter study is necessary to clarify the role of TAT as a potential biomarker for patients with CSF. Thirdly, the absence of longitudinal follow-up data precludes assessment of TAT's prognostic utility for predicting adverse cardiovascular outcomes like myocardial infarction or sudden cardiac death. Future prospective studies with extended follow-up periods are essential to evaluate whether baseline plasma TAT complex levels correlate with long-term clinical trajectories in CSF patients.

## Conclusions

This study presents the first clinical evidence establishing elevated plasma TAT complex levels as a candidate biomarker for CSF. These results advocate for the integration of plasma TAT complex levels into standardized CSF diagnostic algorithms as a cost-effective, noninvasive screening tool.

## Data Availability

The original contributions presented in the study are included in the article/Supplementary Material, further inquiries can be directed to the corresponding authors.
